# Achievements in the Topographic Design of Commercial Titanium Dental Implants: Towards Anti-Peri-Implantitis Surfaces

**DOI:** 10.3390/jcm8111982

**Published:** 2019-11-14

**Authors:** Gerardo Asensio, Blanca Vázquez-Lasa, Luis Rojo

**Affiliations:** 1 Instituto de Ciencia y Tecnología de Polímeros, Consejo Superior de Investigaciones Científicas, CSIC, 28006 Madrid, Spain; gerardo.asensio@ictp.csic.es (G.A.); bvazquez@ictp.csic.es (B.V.-L.); 2 Consorcio Centro de Investigación Biomédica en Red de Bioingeniería, Biomateriales y Nanomedicina, CIBER-BBN, 28029 Madrid, Spain

**Keywords:** dental implants, peri-implantitis, bioactive surfaces, titanium

## Abstract

Titanium and its alloys constitute the gold standard materials for oral implantology in which their performance is mainly conditioned by their osseointegration capacity in the host’s bone. We aim to provide an overview of the advances in surface modification of commercial dental implants analyzing and comparing the osseointegration capacity and the clinical outcome exhibited by different surfaces. Besides, the development of peri-implantitis constitutes one of the most common causes of implant loss due to bacteria colonization. Thus, a synergic response from industry and materials scientists is needed to provide reliable technical and commercial solutions to this issue. The second part of the review focuses on an update of the recent findings toward the development of new materials with osteogenic and antibacterial capacity that are most likely to be marketed, and their correlation with implant geometry, biomechanical behavior, biomaterials features, and clinical outcomes.

## 1. Introduction

The implantation of dental implants has become a common treatment for the replacement of missing or damaged teeth due to the great acceptance of implant therapies, with an estimated placement of more than two million implants per year worldwide, and a tendency to increase its use is expected due to the longer life expectancy of the population [1]. Modern oral implantology includes a large number of devices with different sizes, lengths, shapes, and thread designs [2,3]. The gold standard substrate material employed is pure titanium or Ti−Al−V alloy due to its high corrosion resistance, biocompatibility, and lightweight properties [4]. However, its biological response can be improved by numerous surface treatments that provide bioactivity and osseointegration [5]. The most widespread manufacturing techniques used to obtain bioactive commercially implants are sandblasting, acid-etching, anodization, plasma spraying, and laser radiation. These treatments modify implants’ topography, varying the values of some properties, such as free surface energy, chemical composition, and roughness, which have been demonstrated to allow fast healing and better osseointegration [6,7].

The use of implants that avoid the loss of the device generally obtains very good results, and reaches almost a 90% success rate after 10 to 15 years of implantation [8]. However, between 5% and 11% of dental implants do not achieve a satisfactory degree of osseointegration in the maxillofacial bone, leading to failure of the implant and alteration of the oral function [9]. This implies physical and economic damage to the patient and the specialist. Therefore, the development of a better strategy to preserve the long-term stability of implants that is easily feasible by industrial processes constitutes a priority that must be addressed from a technological and innovative perspective.

Failures of dental implants are usually due to simultaneously occurring biological and biomechanical issues. A notable cause of mechanical failure is the stress shielding caused by the elimination of the typical stress transmitted by the tooth to the supporting bone [10]. When no occlusal force is transferred to the bone by the implant, the bone density is altered by resorptive phenomena and it becomes less dense and weaker [11], which eventually leads to the generation of microfractures and deformations in the surrounding bone with the consequent loss of the implant [12,13]. Another cause of biomechanical failure is an overload applied to the interfacial bone originating from diverse phenomena, such as clenching, excessive cantilevers, inadequate control of wear over time, or bruxism, among others. These conditions are mainly caused by external aspects to the implants coming from either the patient or the specialist. However, biological failures are mostly associated with the accumulation of microbial plaque and bacterial infections, generally known as peri-implantitis, in which the implant design plays an important role.

When a peri-implantitis infection is diagnosed, there are several therapies to save the implant and prevent its removal [14]. In each case, the therapy is chosen mainly according to the extent of the infection. When local antimicrobial therapies fail, it is necessary to use surgical therapies, which involve the resection of the affected tissues, extraction, and decontamination of the surface, followed by a bone graft, applying, in many cases, barrier membranes to prevent epithelial migration [15]. This process generally takes several months, causing oral dysfunctionalities to the patients that cannot be avoided. Thus, in order to prevent the development of peri-implantitis, the use of implants made from materials that enhance the osseointegration processes with the host tissue and at the same time prevent or suppress bacterial colonization is needed. These materials have different routes of action. On the one hand, they promote the formation of bone tissue favoring the activity of osteoblasts to the detriment of osteoclasts or even the differentiation of stem cells towards osteogenic phenotypes. On the other hand, their surface properties and composition may interfere with bacterial adhesion and viability by modifying the surface energy, or releasing antibacterial agents, such as ions, antibiotic drugs, or antiseptics [16,17].

**Figure 1:**
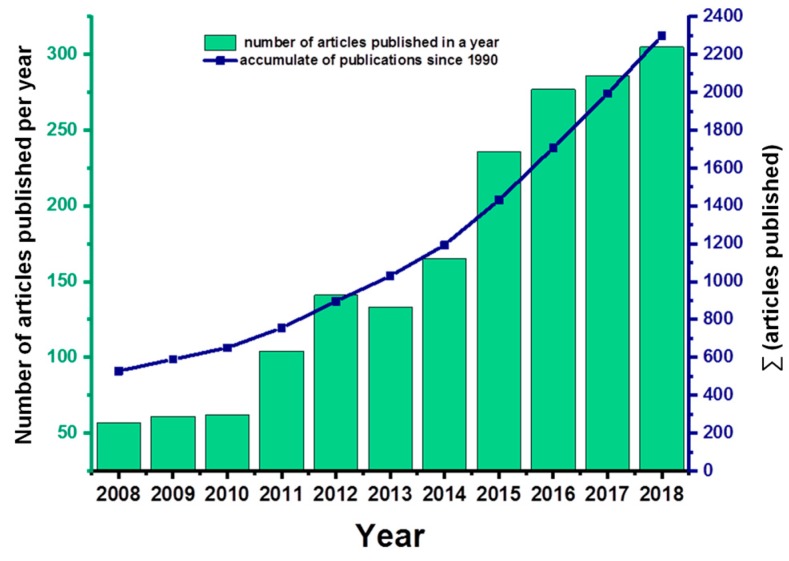
Increasing tendency of dental implant materials research to avoid peri-implantitis disease according to the Web of Science—Thomson Reuters^®^ (Toronto, Canada) database using ‘peri-implantitis’ as the key word.

Thus, a huge effort from the industry and research institutions has been carried out to provide an ultimate solution for all these limitations. In this sense, an increasing number of publications and technical developments have been published claiming new alternatives that combine the ability to prevent bacterial adhesion and biofilm formation with osseointegration capacity and the development of the biological seal (Figure 1). However, no material has been found yet that clearly demonstrates its complete clinical success for peri-implantitis diseases.

In the first part of this review, we summarize the clinical outcomes of current marketed dental implants, describing the correlation between the osseointegration ability of implants and their different surface topography obtained by the manufacturing technique. In the second part, we address the current trends that are closer to becoming the future of oral implantology. Therefore, we recapitulate the most promising recent developments and expect to provide an updated overview of this hot topic issue while at the same time, inspiring future researchers towards the development of new materials applicable in the field of anti-peri-implantitis dental implants. It is worth highlighting that the dental implant brands referred to in this review mainly respond to the availability of peer-reviewed literature reporting advanced in vivo studies correlated with implant geometry, biomechanical behavior, biomaterial features, and clinical outcomes.

## 2. Current Osteogenic Strategies in Implantology

One of the key features that dental and orthopedic implants must fulfill is the capacity of osseointegration with the host bone tissue, allowing a long-lasting and good mechanical performance. To achieve this, commercial implants have been fabricated with topographic and/or physicochemical modification of the surface to confer them the ability to induce a biological response and accelerate the bone regeneration process. Specifically, surface modifications influence the primary interfacial reactions that take place between the implant and components of the blood, connective tissues, and surrounding cells [18,19]. After implantation of the device, its first contact is with the blood generated as a result of bone trauma. A blood clot rich in fibronectin is formed, acting as a scaffold to support the cells of the new tissue [20]. Afterwards, the new bone growth is initiated by osteogenic cells accumulated in the blood clot that secrete a mineralized collagenous interfacial matrix on the implant surface. Finally, bone remodeling takes place at discrete sites and a bone–implant interface is created, comprising new bone that forms [21]. In this sense, it has been found that hydrophilic surfaces promote an up-regulation of angiogenesis-associated genes even at the early stages of bone healing [22,23,24] and support the formation of a more resistant blood clot that cannot be easily disintegrated [25]. Therefore, the chemical composition of the implant and the presence of grafted bioactive molecules and coatings determine important properties, such as free surface energy and surface wettability, which are related to protein adsorption or blood clothing [26,27,28]. In this way, a hydrophilic surface favors a strong blood clot anchorage and ensures that the interfacial bone formed is bonded to the implant surface, promoting a fast osteointegration process from the first day of implantation [29].

In addition, in order to commercialize an implant, it is crucial to consider that the industrial scaling up of the product must be affordable and easily reproducible so that the production process is economically profitable. Most dental implants available on the market have been modified by physical methods to confer roughness on the implant surface. Rough surfaces act as a cellular support, promoting better adhesion and proliferation of osteoblasts in the initial stage of osseointegration due to the increased hydrophilicity [30,31]. As a result, the healing bone process is accelerated, and a stronger device–host bone interface is formed [32]. However, a high surface roughness has been reported as a risk factor in the development of peri-implantitis [33].

Other commercial implants have a ceramic coating that is rich in calcium phosphate (CaP), like hydroxyapatite (HAp) [34]. These inorganic coatings have been extensively studied as an osteoconductive approach due to their similar composition to the mineral component of the bone, fostering the formation of tight bonds with new growing bone [35]. After implantation, the release of calcium phosphate from the coating saturates the biological fluids, forcing the precipitation of an apatite layer on the implant surface [36]. Apatite plays an important role in the healing process as it supports osteogenic cells and may contain endogenous proteins [37]. Moreover, in addition to rough surfaces, the hydrophilicity of CaP and HAp coatings is regarded as being favorable for the initial biological response in blood contact [38,39].

The following subsections review some of the most representative commercially available dental implants, which are classified according to the instrumental technique used to confer different topographies and/or inorganic coatings, as well as their exhibited clinical outcomes.

### 2.1. Macro and Microroughness Surfaces

**Figure 2:**
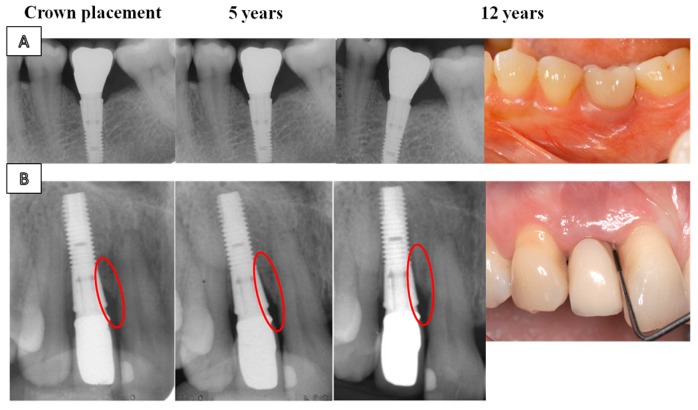
Clinical and radiographic illustrations of: (**A**) A case with no bone loss. Baseline (crown placement), 5 and 12 years of follow-up. (**B**) A case with progressive bone loss. Baseline (crown placement), 5 and 12 years of follow-up. Adapted from Donati et al. [45] with permission from John Wiley and Sons.

A large number of the dental implants that are currently commercialized, as summarized in Table 1, have been obtained using the sandblasting technique [31,40,41]. With this method, it is possible to create a macroroughness by the projection of particles of TiO_2_, Al_2_O_3_, SiO_2_, or HAp generally, which are accelerated with an air stream under pressure to the implant surface. Besides, it should be noted that the incorporation of these particles represents an additional benefit in the development of new bone tissue since they have been reported to be boosting agents of the osseointegration process [42,43], and their effect as an osteoinductive coatings is reviewed further on. The bone–implant interactions exhibited by the different TiO_2_, Al_2_O_3_, and SiO_2_ particles were analyzed in an animal study with sheep [44]. Interestingly, the osseointegration capacity of the different groups was equivalent, despite the implants impregnated with Al_2_O_3_ reaching the highest values of removal torque in the biomechanical test. Concerning the TiOblast^®^ (Astra Tech, Mölndal, Sweden) mark, there are studies in the literature that certify a high success of acceptance after surgical implantation, with acceptable rates of patients affected by mechanical failure, bone loss [45,46], and development of peri-implantitis (3.5% of implants). Figure 2 displays a radiographic illustration of patients with and without bone loss [46]. In a recent long-term study, it was analyzed whether there was an additional benefit in the osseointegration process by comparing implants with a non-modified turned surface (Brånemark Standard Implants^®^ (Nobel Biocare, Gothenburg, Sweden)) and TiOblast^®^ devices [47]. Results suggested that roughness did not improve bone integration, and the authors concluded that there was a lower percentage of implant failures due to peri-implantitis for the non-modified devices, with a total amount of 10.9% of implants affected by this injury. Similar results were published in another comparative study between both types of implants, in which 11% of implant failures were attributed to peri-implantitis disease [48].

The acid-etching methodology can create microrough surfaces by the formation of micro wells on the implant surface as a result of aggressive acid treatment (with acids like HCl, H_2_SO_4_, HNO_3_, and HF, and their combinations) [31,40,41]. Commercial implants manufactured using this technique are registered in Table 1. The ability of acid-etched surfaces to induce an early response in bone regeneration has been studied by comparing the results of turned implants with those of acid-etched surfaces [49]. In this clinical study carried out on 44 people, a larger bone-to-implant contact surface was observed, but no statistically significant differences were reached between each type of implant. Reports provided by the manufacturer stated the correct function of the Osseotite^®^ (Zimmer Biomet, Warsaw, Indiana, USA) product, registering high success rates (>96%) [50,51]. Moreover, comparative studies between non-modified turned surfaces and Osseotite^®^ implants did not show a significant increase in the success rate [52], although removal torque values were four times greater for Osseotite^®^ implants [53]. The long-term stability of Swede-Vent^®^ (Zimmer Biomet, Palm Beach Gardens, Florida, USA) and Screw-Vent^®^ (Zimmer Biomet, Palm Beach Gardens, Florida, USA) implants, produced by the same acid-etching process but a varied screw length, was compared with non-modified Brånemark^®^ implants in a clinical study [54]. By histomorphometric analysis it was observed that all three designs achieved great long-term stability, noticing also that Brånemark^®^ together with Swede-Vent^®^ implants presented the lowest bone loss, reaching significant differences compared to Screw-Vent^®^, and suggesting that a longer screw length is associated with a greater bone loss.

**Figure 3:**
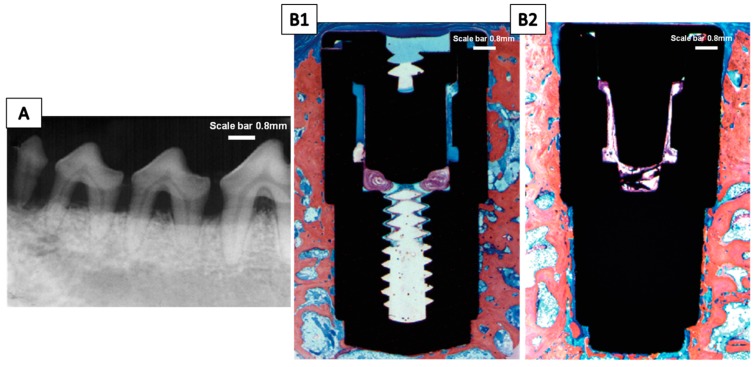
(**A**) Radiographic aspect of the teeth after 3 months of disease induction. Note the bone loss at the furcation and proximal areas, indicating the presence of periodontal disease. (**B1**) Bone–implant contact of the grit-blasted/acid-etched surface (original magnification ×12.5, Stevenel’s blue and Alizarin red stain). (**B2**) Bone–implant contact of the titanium plasma spray surface (original magnification ×12.5, Stevenel’s blue and Alizarin red stain). Figure adapted from Novaes Jr. et al. [60] with permission from John Wiley and Sons.

The roughness of the implants can be increased by applying an acid treatment after particle blasting. Thus, the formation of a complex topographic geometry takes place at the micrometric level: The macroroughness created by the projected particles is combined with the microroughness caused by acid etching [55]. The name of the entire process is known as sandblasting and large grit acid etching (SLA). This method has been reported as a promising technique to confer biological activity to the implants’ surface due to an increase in surface energy and wettability, which positively contributes to the bone healing process in the initial stages [56]. Histomorphometric analyses of these surfaces showed a high level of bone-to-implant-contact [28,57]. In this context, implants available in the market obtained by this methodology are collected in Table 1. SLA Straumann^®^ (Straumann Institute, Basel, Switzerland) devices are an illustrative example of the surfaces obtained by this methodology. In one study, these implants were evaluated and compared with an acid-etched surface using an animal model. Removal torque measurements were carried out by means of interfacial stiffness calculation, in order to evaluate the interlocking degree between the implant and the host tissue. Data revealed that a small increase in this value was registered for SLA Straumann^®^ surfaces, but the differences were not statistically significant [58]. Friadent Dentsply (Mannheim, Germany) surfaces are characterized by a dynamic change in the wettability surface ratio. It moves from an initial hydrophobic state to a hydrophilic state when the implant is in contact with extracellular matrix proteins [27]. A clinical study examined the effect of early or late loading on devices manufactured by Friadent Dentsply with an SLA surface. The results obtained showed that there were no significant differences between the Friadent Dentsply products, reaching a high overall success rate (>99%) in terms of the absence of pain and peri-implant infection mobility and peri-implant bone resorption [59]. Furthermore, another study with an animal model revealed that a high percentage of bone-to-implant-contact was reached after 12 weeks of healing even when they were inserted into a periodontitis-induced dog model (Figure 3) [60].

Anodization is another technique used to obtain microtopographies. It is based on a potentiostatic or galvanostatic electrochemical oxidation of titanium surfaces using strong acids, such as H_2_SO_4_, H_3_PO_4_, HNO_3_, or HF, creating a uniform porous layer of TiO_2_ in the form of anatase, which is thicker than the initial one [31,40,55]. Under certain conditions, the anodization technique can create a porous organized surface in the form of TiO_2_ nanotubes (TNTs). These kinds of nanostructured surfaces and their functionality will be described below in Section 3.1. The anodization treatment increases blood clot retention, which favors the osseointegration of the device [61]. The chemical modification of the surface not only provides a significantly greater increase in bone response but also better biochemical adhesion due to the interconnection of new bone tissue through the pores of the implant [62]. A representative commercial implant of this group includes the brand TiUnite^®^ (Nobel Biocare, Gothenburg, Sweden). Highly successful rates have been reported in prospective studies for these implants (>96%) [63,64], of which 8.2% of implants were positive for peri-implantitis disease [63]. However, no significant differences in cumulative survival rates were reached when compared with a non-modified turned surface [65,66]. Moreover, the biomechanical properties of TiUnite^®^ have been evaluated against acid-etched surfaces provided by Osseotite^®^ and oxidized magnesium surfaces. The results showed that there were no significant differences between the three groups, and TiUnite^®^ implants exhibited an intermediate value in the removal torque [67].

**Figure 4:**
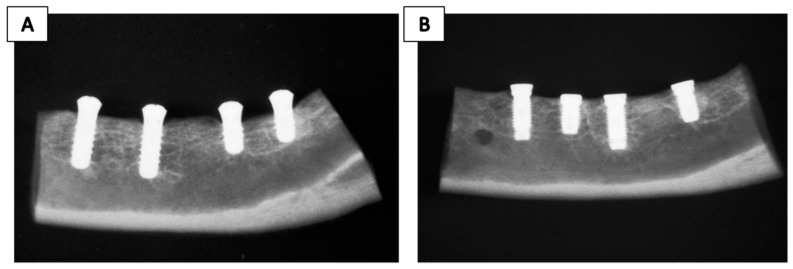
Radiograph taken after mandibular resection. Note that implants are embedded in the surrounding bone; no radiolucency was detected. (**A**) Bone segment with Brånemark implants, (**B**) bone segment with ITI-TPS^®^ commercial implants. Reproduced from Bernard et al. [71] with permission from John Wiley and Sons.

Other methods for implant surface modifications exist, such as plasma spraying, which modifies the topography of the implants by the projection of a Ti powder injected into a plasma torch at high temperatures, forming a film about 30 µm thick constituted by Ti-OH groups [31,40,55]. The process can be accomplished with atmospheric or reduced pressure and has the advantage of not leaving chemical residue traces on the surface, which happens with wet techniques [40]. Recently, the capacity of SLA surfaces modified with the plasma spraying treatment was demonstrated to potentiate cellular adhesion and the adsorption of proteins due to the generation of a layer of OH groups that increase the superficial hydrophilicity [68,69]. Implants manufactured by the titanium plasma spraying (TPS) technique are included in Table 1; all techniques are produced at atmospheric pressure to reduce industrial production costs. The ITI-TPS^®^ (Straumann Institute, Waldenburg, Germany) product achieved satisfactory results in terms of long-term stability, bone-to-implant-contact, resorption of marginal bone, and removal torque values [34,70]. In a canine model study, it was reported that values of the removal torque and marginal bone loss reached significant differences for ITI-TPS^®^ implants compared with non-modified surfaces [71]. Figure 4 represents a radiographic illustration of both implants after mandibular resection. Moreover, different histomorphometric analyses performed in a dog model showed that ITI-TPS^®^ implants (rough surface) experience a slight benefit from bone-to-implant contact compared with Brånemark^®^, TiUnite^®^ and SLA Straumann^®^ implants (minimally rough surfaces) [72], and also successful osseointegration even when they were implanted into mandibular bones affected by periodontitis (Figure 3) [60].

### 2.2. Nanoroughness Surfaces

To optimize the surface roughness of the implant, its topography has been modified at the nanometric scale (1–100 nm) in a few commercial brands. The nanotopographic design of the surface recreates the natural cellular environment, mimicking the implant with the native tissue. This implies the generation of cellular interactions, triggering changes at the physical, chemical, and biological levels, which lead to an increase in the attachment of adhesive proteins and osteoblasts to the surface [73], thus promoting a higher bone-to-implant interface and accelerating device integration [74]. Table 2 shows some nanorough surface implants according the topographical characteristics found in previous studies [75].

A simple method for making nanotopographies is the laser ablation [40,76] technique, which is employed in the product Laser-Lok^®^ (BioHorizons, Birmingham, Alabama). This implant has the advantage of increasing the interconnection of the surface with fibrous and soft tissues, since in the manufacturing process the imparted nanotopography is applied to the neck of the implant. The use of laser micromachining generates micro and nanochannels on the surface that serve as support for cells, promoting fast osseointegration and, at the same time, high attachment of soft tissues and low epithelial growth [77,78]. In a prospective canine model study, histological analysis revealed that there were significant differences in the bone-to-implant-contact value when the Laser-Lok^®^ surface was compared with a conventional machine-turned surface. In addition, this study also confirmed a high degree of new hard and soft tissue growth around the implant [79]. Similar results were found in a prospective clinical study in which the authors found that the implantation of Laser-Lok^®^ surfaces resulted in improvements in the clinical attachment level versus turned non-modified surfaces, and also a significantly lower crestal bone loss grade [80]. Additionally, another study with an animal rabbit model compared the mechanical stability of laser ablation-modified prostheses (using a neodymium-doped yttrium aluminium garnet laser, Nd-YAG) and TiUnite^®^ anodized surfaces, obtaining a significantly higher torque value for the laser-treated surfaces [81]. Interestingly, a recent study has shown that laser treatment after the SLA process does not make a notable difference in the osseointegration process [82].

**Figure 5:**
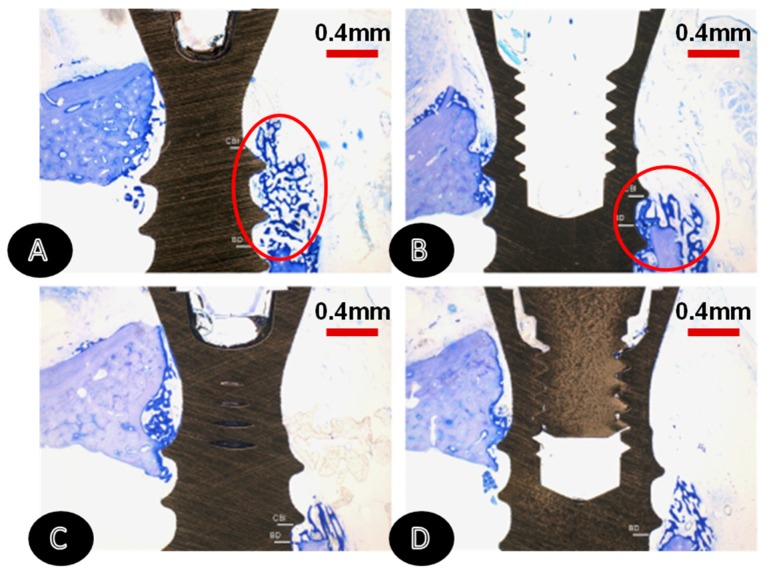
Representative histological views of wound healing in different groups at 2 weeks. Bone regeneration in close contact with the implant surface was most pronounced at the lateral aspect of SLActive^®^ commercial implants (upper jaws, TB stain, original magnification ×25). (**A**) SLActive^®^, submerged, lateral aspect. (**B**) SLActive^®^, submerged, central aspect. (**C**) SLA Straumann^®^, submerged, lateral aspect. (**D**) SLA Straumann^®^, submerged, central aspect. Figure adapted from Schwarz et al. [87] with permission from John Wiley and Sons.

SLActive^®^ (Straumann Institute, Basel, Switzerland) is another dental implant trademark based on a surface with nanoscale geometry. These implants are manufactured by grit blasting and acid etching following the same process used for SLA Straumann^®^ surfaces, but including a final stage in which the implant is flushed with a stream of nitrogen to avoid contact with air and then instantly introduced into an NaCl solution for storage. SLActive^®^ surfaces are characterized by high hydrophilicity, which favors the induction of a biological response in the host tissue and thus contributes to the formation of a homogeneous new bone layer around the implant as a consequence of increased adhesion of fibrinogen and fibronectin in the early stages of healing [55]. Moreover, early blood coagulation processes are favored by the increased hydrophilicity and positively affect the osseointegration process [83]. This proves that SLActive^®^ surfaces are closely related to the differentiation mechanisms of osteoblasts, fostering recruitment of these cells [84]. The mean average survival rate reported for SLActive^®^ surfaces was high (98.5%), as well as for its analogue Roxolid^®^ (Straumann Institute, Basel, Switzerland) (96.8%), whose substrate is made of a titanium–zirconium alloy [85]. The long-term durability of SLActive^®^ implants was further analyzed in a prospective study, which concluded that the survival rate was acceptable at 91.7% and that the degree of marginal bone resorption was very low, with 2.5% of implants affected by peri-implantitis, suggesting that a strong physical interaction takes place in successful devices [86]. Interestingly, histomorphometric analysis of SLActive^®^ implants performed in a canine model indicated additional benefits in the healing and regeneration of new bone tissue compared to its predecessor, SLA Straumann^®^ (Figure 5) [87].

The Osseospeed^®^ (Dentsply Friadent, Mannheim, Germany) brand with nanometric roughness is produced by an acid treatment with HF once the blasting of TiO_2_ particles on the surface is carried out. In a prospective study with a three-year follow-up, with implants installed in healed ridges or extraction sockets, it was concluded that both hard and soft tissues were positively stabilized and their survival rates were comparable regardless of the site of implantation [88]. Clinical and radiographic examinations published in another prospective study with a one-year follow-up revealed that marginal bone loss was minimal [89]. Besides, other authors have analyzed whether the post fluoridation process of the Osseospeed^®^ product provides enhancement of the bone-to-implant-contact when compared to TiOblast^®^ implants. However, histomorphometric and histological analyses of five implant pairs revealed that Osseospeed^®^ nanotopography did not guarantee greater osseointegration [90].

### 2.3. Inorganic Coatings

Commercial dental implants can also be coated with inorganic compounds, with the aim of accelerating early bone healing. The most commonly used coatings are nanostructured calcium, CaP, and HAp, and their incorporation is mainly achieved by hydrotermal deposition [91] or the plasma spraying technique [40,55]. As they are a source of calcium and phosphate ions, it is expected that they will give rise to a more mineralized bone–implant interface. The debate about the advantages of inorganic coatings during their in vivo performance is still open in the literature. On the one hand, some researchers have reported that implants with CaP coatings present a higher percentage of bone-to-implant contact compared to non-coated implants, which confers a high adhesion strength to the host bone [92,93]. On the other hand, other authors support that the presence of HAp on the surface increases the risk of bacterial adhesion and colonization of periodontal pathogens along with the level of crestal bone resorption and mobility of the implant [94,95]. Nevertheless, implants with CaP coatings fabricated by plasma spraying evidence a weak increase in the osseointegration process, pending more clinical trials evaluating the different properties conferred to implants when they are produced by other techniques [96].

**Figure 6:**
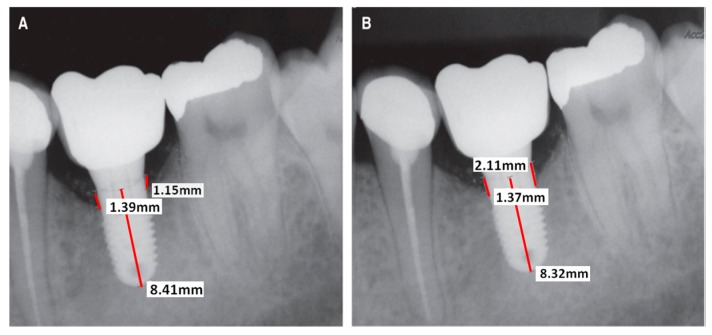
Example of measurement using periapical radiographs. (**A**) Left radiograph was taken after delivering a prosthesis, (**B**) Right radiograph was taken during the follow-up period. Both radiographs are Zimmer^®^ commercial implants in the same patient. Figure adapted from Cervino [99].

Examples of commercial implants with an inorganic coating are collected in Table 3. It seems that the presence of the inorganic coatings can affect the transmission of mastication forces from the bone to the implant. In this sense, the way in which Osstem GS-HA III^®^ and Osstem TS III-HA^®^ (Osstem Implant Co., Busan, Korea) implants transmit the stress produced in mastication to the host bone tissue was analyzed [97], finding a correct distribution of the simulated force that is applied in the masticatory cycles. However, further studies on the same type of implants using finite element method (FEM) analysis revealed that to ensure their long-term use, it is still necessary to develop a more advanced prosthetic balance that homogeneously distributes stress [98]. The Osstem TS III-HA^®^ and TSV-HA^®^ (Zimmer Biomet, Carlsbad, California, USA) products were compared and both exhibited high survival rates, but significant differences in terms of the healing process were achieved after immediate loading (Figure 6). Moreover, peri-implantitis was diagnosed in 2.2% of Osstem TS III-HA^®^ implants and in 1.4% of TSV-HA^®^ implants [99]. These models were also evaluated in a retrospective analysis, which reported a survival rate success of 100% and no differences between the two products [100].

**Figure 7:**
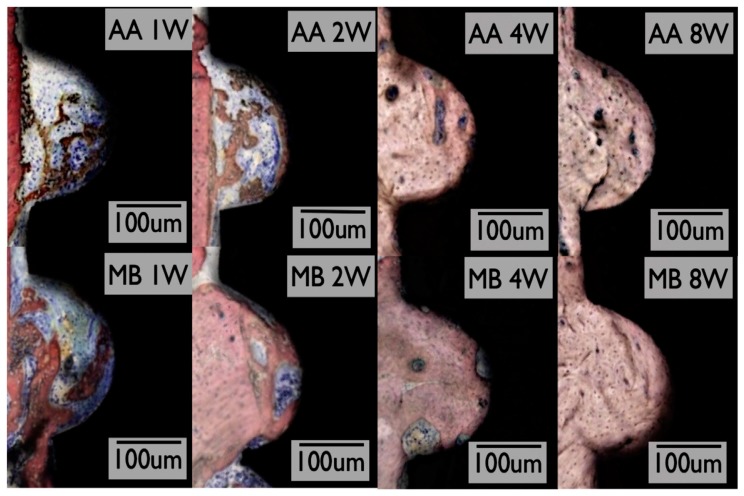
Histological sections for Ossean^®^ (MB) and dual acid-etched (AA) groups at 1, 2, 4, and 8 weeks in vivo. Reproduced from P.G. Coelho et al. [101] with permission from Elsevier.

In the case of the Ossean^®^ (Intra-Lock, Boca Raton, Florida, USA) brand, the biomimetic nanotopographic design is combined with the action of CaP as a mineral component. Its manufacturing process consists in the impregnation of nanometric-sized particles of calcium phosphate into the implant surface, which has been previously subjected to grit blasting and subsequent acid etching. The result of the process is the formation of a nanoroughness by the deposition of the CaP nanoparticles within a microroughness formed by the grit blasting/acid-etching process. To better understand the cell behavior induced when exposed to the nanotopography of Ossean^®^ implants, the gene expression of the most relevant osteogenic markers was measured and implants with a microroughness obtained by double acid etching (AA group) were used as the control group [101]. The real-time quantitative reverse transcription polymerase chain reaction (RT-PCR) revealed a significant increase in the regulation of osteogenesis-related genes after 1 and 2 weeks post-implantation. Moreover, histomorphometric analysis showed that there was a high mineralization of bone tissue around Ossean^®^ implants (MB group), reaching significant differences with respect to the dual acid-etched surfaces used as the control (AA group, Figure 7). In another study, the activity of osteoblast-like human osteosarcoma cells (SaOS-2) and bone mesenchymal stem cells on the surface of Ossean^®^ implants was evaluated [102]. Significantly greater alkaline phosphatase (ALP) kinase expression was observed at all measured times for Ossean^®^ implants, and acceptable values in terms of adhesion and cell proliferation were obtained.

The acid-etching method can be employed in combination with a subsequent sol-gel process called discrete crystalline deposition (DCD) during manufacturing of nanostructured surfaces with inorganic compounds [103]. In the case of the brand Nanotite^®^ (Zimmer Biomet, Palm Beach Gardens, Florida, USA), nanotopography is formed by the biomimetic deposition of nanometric particles of CaP in the DCD treatment. The nanostructured surface of these implants has been reported to be an effective osteoconductive surface [104,105]. In a clinical trial, these implants showed a favorable mean survival rate, as well as a low degree of bone loss around the implant [106]. Another study with an animal model revealed that there were significant differences in the bone-to-implant contact value between the nanogeometry and microtopographic surfaces [105]. The same result was reported in another comparative study between Nanotite^®^ and Osseotite^®^ products with nano and micro roughness surfaces, respectively [107]. The biomechanical properties shown by Nanotite^®^ implants have also been tested in a canine animal model in which they evaluated four surfaces obtained by different methods, micro blasted, acid etched and micro blasted, anodized, and discrete crystalline deposition, concluding that only acid-etched implants presented a significantly higher removal torque value than DCD surfaces [108]. On the contrary, other authors reported that the presence of CaP nanocrystals on the surface of the implant involved an improvement in the anchorage of the implant to the bone matrix [103].

### 2.4. Bacteria Colonization

The process by which implants undergo bacterial colonization has been extensively studied to improve biomaterials’ performances. In the oral cavity, a range of more than 500 different bacteria coexist, of which only 300 have been named [109]. Like osteogenic cells, these bacteria are susceptible to adhering to the implanted biomaterial and a ‘race to the surface’ takes place between them [110]. In the early stages of colonization, plankton bacteria attach to the surface of the implant by van der Waals or gravitational forces. After this deposition, the bacteria associate with each other with greater strength through various mechanisms specific to each microorganism, such as flagella, pili, proteins, and polysaccharide adhesions [111]. This process is accentuated for dental implants by the presence of bacterial plaque in the mouth [112]. Small aggregates of these bacteria begin to secrete an extracellular matrix rich in polysaccharides and proteins until a resistant biofilm is formed.

Biofilm acts as a refuge for bacteria by protecting them from the host immune system and antibiotic therapies, and can lead to the development of a permanent infection that persists even under aggressive antibiotic treatment [113,114]. Besides, osteoblasts can absorb bacteria and store them in vesicles. However, bacteria have developed defensive mechanisms by which they release toxins that allow them to escape from internalization and cause necrosis and apoptosis to the host’s osteoblasts. In addition, other neighboring osteoblasts will be infected by these bacteria, producing a cascade of proinflammatory molecules, like cytokines and chemokines [115]. By this way, the osteoclastogenesis process is potentiated, leading to bone resorption as a consequence of an imbalance between osteoblasts and osteoclasts [116,117].

Additionally, during the host response to a bacterial infection, complementary activation from the innate immune system takes place [115]. Inflammatory cells are attracted to the infected site and proinflammatory cytokines are produced, which further boost osteoclastogenesis [118]. In sum, the immune system inflammatory reaction could be triggered by the presence of metal particles, cement residues, and organic contaminants non-covalently bonded to the surface [119]. These results become a problem when they exceed more than a 10-µm size or are high in numbers because macrophages are unable to phagocytize them and an overproduction of proinflammatory cytokines around the implant takes place, thus increasing the risk of developing peri-implantitis [120,121].

After bacterial colonization and biofilm formation, the risk of implant failure is enhanced by the development of diseases, such as peri-implantitis, resulting in enormous economic costs and physical and emotional damage caused to both the patient and the specialist [122]. Thus, peri-implantitis is described as an irreversible inflammatory process of the tissues around the dental implant, which starts with a localized inflammation and entails the destruction of soft tissues and loss of peri-implant bone [14]. The diagnosis criteria for peri-implantitis disease have recently been established in the World Workshop on the Classification of Periodontal and Peri-implant Diseases and Conditions, aiming to clearly provide a reliable consensus between periodontitis, peri-implantitis, and peri-implantitis mucositis pathogenesis [123]. It is worth mentioning that despite periodontitis and peri-implantitis having some similarities in terms of pathogenesis and therapy, their etiology differs in that the origin is the presence of an implant [124].

In most of the published studies in which implant performance was evaluated, peri-implantitis development is not reported or the diagnostic criterion is unclear or does not follow a quality consensus as described above [123]. An effort has been made by researchers to estimate the proportion of dental implants affected by peri-implantitis. A systematic review and meta-analysis reported that the frequency of peri-implantitis development is 18.8% of patients and 9.6% of implants out of 504 studies with 1497 participants involved [125]. In a systematic review, 25 randomized controlled trials (RCTs) were analyzed, aiming to identify whether superficially modified implants pose a greater risk of peri-implantitis development than non-modified turned implants [126]. It was found that despite not reaching statistically significant differences, implantation of non-modified implants reduces the risk of peri-implantitis development by 20%. However, this result must be interpreted with caution because it was extracted from four RCTs with short follow-up periods (≤3 years). Another recent systematic review, including prospective and retrospective studies, evaluated the resistance of modified and non-modified implants to exhibit long-term peri-implantitis resistance (≥5 years) [127]. Considering the heterogeneity of the included studies, again, it was observed that there were no significant differences between the two types of surfaces and that less than 5% of the implants were positive for peri-implantitis disease.

Once peri-implantitis has been diagnosed, a treatment to decontaminate the surface is necessary because the presence of this condition does not strictly imply a loss of the implant. Several methods for the treatment of this condition have been reported with favorable results [128]. Among them, the use of an erbium-doped yttrium aluminium garnet laser (Er-YAG), mechanical debridement, alone or with local application of antibiotics or antiseptic agents, the application of a bone substitute (nanocrystalline hydroxyapatite), and regenerative membranes stand out [128,129]. Since no methodology has shown a clear superiority over the other, the best way of action is the prevention of bacteria colonization by the design of new implants with antimicrobial surfaces.

## 3. Current Trends: Osteogenic Coatings with Antibacterial Potential

Surgical implantation of orthopedic and dental implants presents the risk of possible bacterial infection and failure of the device, as a result of an immune response triggered by the presence of a foreign body in the organism, and furthermore, the adhesion and fixation of bacteria is favored by the use of implants with rough surfaces [33]. Commercially available titanium implants present important differences in their surface microstructure and topography depending on the manufacturing technique used and the presence of coatings, as summarized in Table 4. Therefore, topographic modifications can generate surfaces that can be classified as a function of their roughness (Sa) in smooth surfaces (S_A_ = 0–0.5 µm), minimally rough (S_A_ = 0.5–1 µm), moderately rough (SA = 1–2 µm), or rough surfaces (S_A_ > 2 µm) [130], and must be combined with antimicrobial agents or anti-adherent surfaces of bacteria to minimize the risk of biofilm formation and ensure its durability [131,132]. Currently, commercially available implants with antibacterial capacity are limited to the field of orthopedic implantology [133]. It should be noted that the oral conditions to which dental implants are subjected (mechanical stress, bacterial strains, etc.) vary with respect to the orthopedic implant. For this reason, thorough research in dental implantology is still needed to satisfy the aesthetic and functional conditions required by implants installed in the oral cavity.

Bioactive agents that prevent bacterial infection can be classified according to the mechanism of action [132], including passive coatings that prevent bacteria from attaching to the implant surface and active systems that release antibacterial agents killing the surrounding bacteria. Passive systems consist mainly of polymeric coatings that decrease the adhesion of cells and proteins. They are an attractive alternative due to the simplicity of their handling, as they do not require the use of drugs. However, the anti-adherent character equally affects the adhesion of osteoblasts to the implant, so the osseointegration process is compromised. To mitigate this adverse effect, antifouling coatings are functionalized with cellular adhesive peptides that favor the cell–implant interaction, enhancing osteogenesis while inhibiting the fixation of bacteria. Concerning active coatings, the agents responsible for killing the bacterial strains (metallic ions, antibiotics, or disinfectants) can be physically adsorbed or embedded in a polymeric matrix (for example, antifouling polymers), as well as being covalently anchored to the coating (antimicrobial peptides). The latter case avoids the problems associated with the drug burst release that can cause toxicity to the osteoblastic cells, as well as loss of the antibacterial effect over time. Some orthopedic implants available on the market have an antibacterial ability. Some examples are those produced with active coatings, such as gentamicin contained in a matrix of poly (D, L-lactide) for tibia nails, a coating of povidone-iodine for titanium implants, and silver coatings, either by galvanostatic deposition of a large amount of elemental silver or by incorporation of a low concentration of silver ions by anodization of the piece in aqueous solution, both for tumor endoprostheses and knee arthrodesis nails [133].

In summary, the incorporation of antibacterial compounds to prevent the formation of bacterial biofilm compromises the viability of osteoblastic cells that form new tissue whereas physico-chemical modifications of the surface favor the fixation of both bacteria and osteoblasts. For this reason, new trends focus on systems combining osteoconductive or osteoinductive compounds, which potentiate the proliferation and differentiation of native cells, with antibacterial agents used to prevent the development of infections. In this regard, both effects can act synergistically, ensuring the success of the entire osseointegration process. Thus, in the following subsections, we review the most outstanding papers in which both aspects are evaluated.

### 3.1. Metal Ions and Nanoparticles

**Figure 8:**
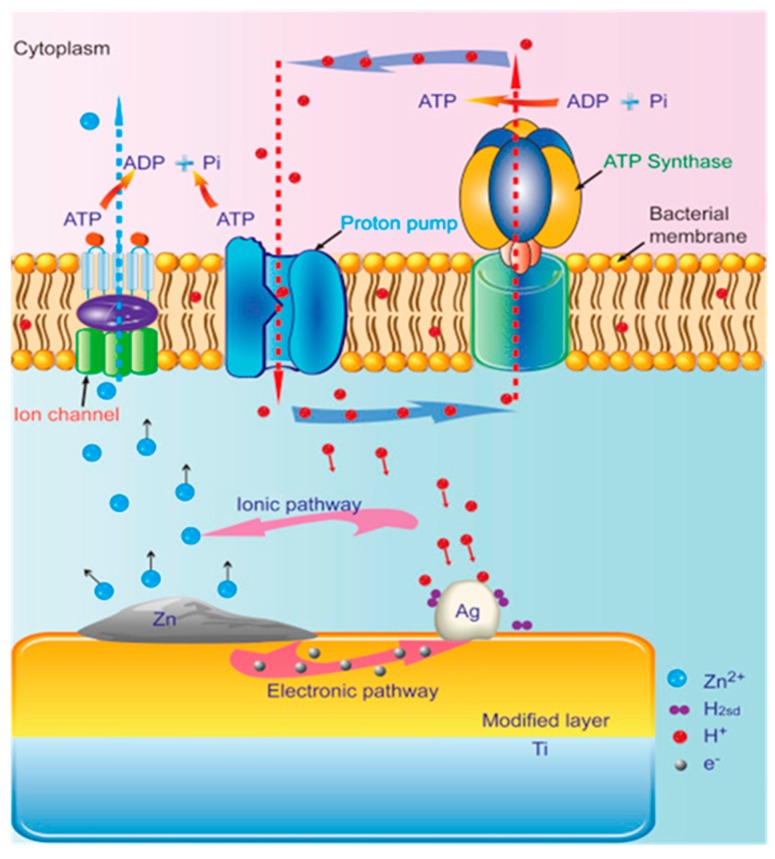
Schematic illustration of the possible antibacterial mechanism on the Zn/Ag co-implanted titanium surface. Reproduced from G. Jin et al. [138] with permission from Elsevier.

The antibacterial capacity of dental implants described in the literature is mainly conferred by ions or nanoparticles of metallic elements. Silver is the most commonly used antibacterial compound [134,135,136,137,138,139,140,141,142,143,144,145], followed by zinc [138,146,147,148,149,150] and copper [151,152], although some authors have reported antimicrobial properties for cerium [153], tantalum [154], titanium [145,155], and magnesium [147]. The high antibacterial capacity of silver has been tested against a large number of bacterial strains. For example, the antibacterial potential of stainless-steel surfaces modified with silver nanoparticles immobilized uniformly by the plasma immersion ion implantation (PII) technique has been tested against different bacterial strains, including gram-positive and gram-negative strains [134]. PII methodology allows a strong fixation of metal compounds, simultaneously avoiding their mobility and the subsequent hazardous effects. Additionally, the same authors studied the effect of silver nanoparticles on the differentiation of human bone marrow stromal cells. The spreading and differentiation of mouse osteoblastic cells line (MC3T3-E1) were enhanced as it was shown by the quantification of ALP expression and extracellular matrix mineralization [135]. An immersion assay in phosphate-buffered saline (PBS) revealed the long-term (75 days) antibacterial capacity of substrates and the ALP activity and extracellular matrix mineralization confirmed a beneficial effect on the spreading and differentiation of osteoblastic cells. The osseointegration ability of Ag nanoparticles immobilized in a titanium SLA dental implant by PII was further investigated in a canine model study with Labrador dogs [136]. The implant stability, micro-computed tomography (micro-CT) assay, histomorphometry, and histological analyses showed good development of both soft and hard tissues around the implant and a greater osseointegration process of Ag PII-modified implants compared to control SLA surfaces. Subsequent studies evaluated the antibacterial capacity of these implants, resulting in a significant growth inhibition of *Fusobacterium nucleatum* and *Staphylococcus aureus* [137]. In view of the data obtained, the authors suggested that Ag PII-modified implants have both osteogenic and antibacterial potential without delamination and side effects. The use of the PII technique has also been reported for the modification of implant surfaces with other ions, like zinc [138,146,147], magnesium [147], both zirconium and nitrogen [156], and their combinations, to obtain a synergistic effect in the successful acceptance of the implant [138,147]. The Zn-implanted titanium showed an advantageous effect on the osteogenic activity and a partial antibacterial activity against *Escherichia coli* and *Staphylococcus aureus* that could be increased with longer implantation times [146]. The co-immobilization of Zn and Ag in Ti implants improved the corrosion resistance of the substrate, enhanced the cell adhesion and spreading activity of rat bone marrow stromal cells (rBMSCs) in the initial phases of osseointegration, and significantly increased the bactericidal power both in vitro and in vivo due to the synergistic effect of the two ions (Figure 8) [138]. The co-immobilization of Zn and Mg in Ti implants not only enhanced the expression of osteogenic markers and showed potential killing of aerobic bacteria but also promoted the manifestation of a magnesium transporter-1 (MagT1), inducing angiogenesis by the action of the Mg^2+^ ion [147].

**Figure 9:**
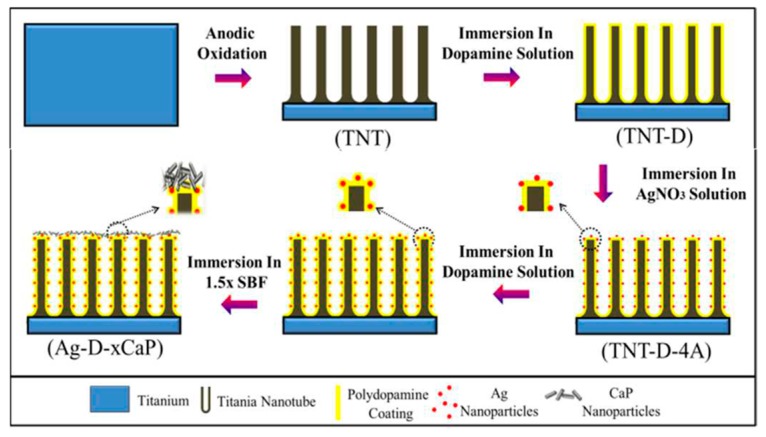
Illustration of anodization, dopamine polymerization, silver reduction, and CaP biomineralization on Ti. Reproduced from M. Li et al. [145] with permission from the Royal Society of Chemistry.

Dental implant coatings based on TNTs are an effective strategy for the development of devices with osteogenic and antibacterial properties. The nanotubular geometry obtained by applying certain conditions in the anodization process has been demonstrated to promote the spreading, differentiation, and attachment of osteoblast cells [86]. In addition, the capacity of nanotubes (NTs) to reduce the colonization of bacteria has been reported, as the nanotopographic design favors osteoblasts versus bacteria adhesion to the surface because of the larger size of the cells [157]. In order to increase the bactericidal capacity of NT, many current researches are focused on the use of these structures as reservoirs of antibacterial agents for their in situ release in the implanted area. In this sense, the formation of TNT decorated with silver nanoparticles [139,140] alone or combined with strontium [141,142], titanium dioxide nanoparticles [145], or silver oxide nanoparticles loaded into Ta NT has been reported [143]. In all cases, the antibacterial ability of the NTs increased due to the presence of the different metal nanoparticles, and besides, all samples showed an enhancement of osteogenic activity. This effect was significantly higher in samples with strontium as it has been extensively described as a promoter of the bone tissue regeneration process [158]. An important aspect of these systems is that the release must be rigorously controlled since a burst release of the antimicrobial agent can lead to mortality of osteoblastic cells. In this sense, several researches are focused on the development of sealing coatings placed over NTs that allow a controlled and sustained drug release over time. This was achieved with a coating made of folic acid coordinated with ZnO quantum dots (QDs), which are unstable in acid environments, allowing the release of vancomycin encapsulated in the TNTs. The antifouling effect of vancomycin was potentiated by the action of ZnO QDs, resulting in an almost complete eradication of *Staphylococcu aureus* while folic acid positively affected the proliferation and differentiation of MC3T3-E1 cells due to its role in DNA replication processes [148]. NTs coated with polydopamina is another method to obtain systems with a controlled release of antifouling agents. Polydopamina (PDA) coatings can be considered as an attractive strategy due to the simplicity of the process (self-polymerization after soaking in a dopamine solution), their good interaction with organic and inorganic substrates, their excellent biocompatibility and biodegradability, and their potential to scavenge free reactive radicals because of its high antioxidant properties. The latter aspect was employed to in situ reduce Ag^+^ to Ag^0^ via catechol oxidation after dopamine polymerization, leading to the formation of a uniform and deep coating of silver nanoparticles [144]. As was expected, Ag nanoparticles efficiently inhibited the colonization of six types of bacteria (*Escherichia coli*, *Staphylococcus aureus*, *Candida albicans*, *Streptococcus mutans*, *Actinomyces israelii*, and *Lactobacillus acidophilus*), however, the attachment, proliferation, and differentiation of osteoblast cells were negatively affected. To overcome this problem, the authors soaked the system in simulated body fluid (SBF) and the activity of osteoblasts was improved by the deposition of CaP on TNTs. Similar results were obtained in another investigation in which, after silver nanoparticle deposition was performed, the system was immersed in a dopamine solution, again to ensure a complete embedment of silver nanoparticles and induce better mineralization (Figure 9) [145]. Furthermore, in other reports, the osteogenic properties of the substrates were improved by the covalent immobilization of an Arg-Gly-Asp-Cys peptide (RGDC) with a dopamine coating that can engage integrin adhesion receptors on cells [149]. In this work, ordered and homogeneous Zn nanorods were formed on Ti substrates by hydrothermal treatment followed by self-polymerization of polydopamine and subsequent bonding of RGDC peptides. The coordination bond between Zn and PDA yielded a controlled and sustained release of metal ions, resulting in an efficacious killing of *Staphylococcus aureus* and *Escherichia coli* and an enhancement of the attachment, proliferation, and differentiation of osteoblasts. 

### 3.2. Bactericidal Peptides

Bactericidal peptides have been reported as an effective bacteria killer with short times of action, a broad spectrum of antimicrobial activity, and low susceptibility to the development of resistant strains. Silanization is a methodology employed to covalently bind bactericidal peptides and antibiotics to titanium surfaces. Thus, the covalent fixation of the anti-inflammatory and bactericidal GL13K peptide on Ti implants has been reported [159]. The authors studied the osseointegration capacity of GL13K peptides through micro-CT scanning and histomorphometry in a rodent model, identifying acceptable levels of regenerated bone tissue with good clinical outcomes of the modified implants and the prevention of microbial infection. In a similar way, other authors have described the anchoring of RGD, KRWWKWWRR (HHC36) and human lactoferrin-derived (hLf1-11) peptides with a silane coupling agent, resulting in excellent inhibition of bacteria adhesion and good biocompatibility [160]. The binding of hLf1-11 peptide via silanization with (3-aminopropyl)triethoxysilane (APTES) resulted in high rates of bacteria being killed [161], and its anchorage onto titanium surfaces has been reported as an osteogenic route by the upregulation of the bone markers measured. At the same time, the adhesion of *Streptococcus salivarius* and *Streptococcus sanguinis* was remarkably reduced without inhibition of eukaryotic cells’ viability grown in co-cultures with fibroblast cells [162]. The physical adsorption of bactericidal peptides, such us streptococcal collagen-like protein and b-defensin 3 (HBD-3) peptides on titanium substrates, constitute other interesting ways to prevent the development of infections, showing rapid early bone regeneration, despite some limitations regarding peptide stability and lifespan [163,164].

### 3.3. Antibiotics 

**Figure 10:**
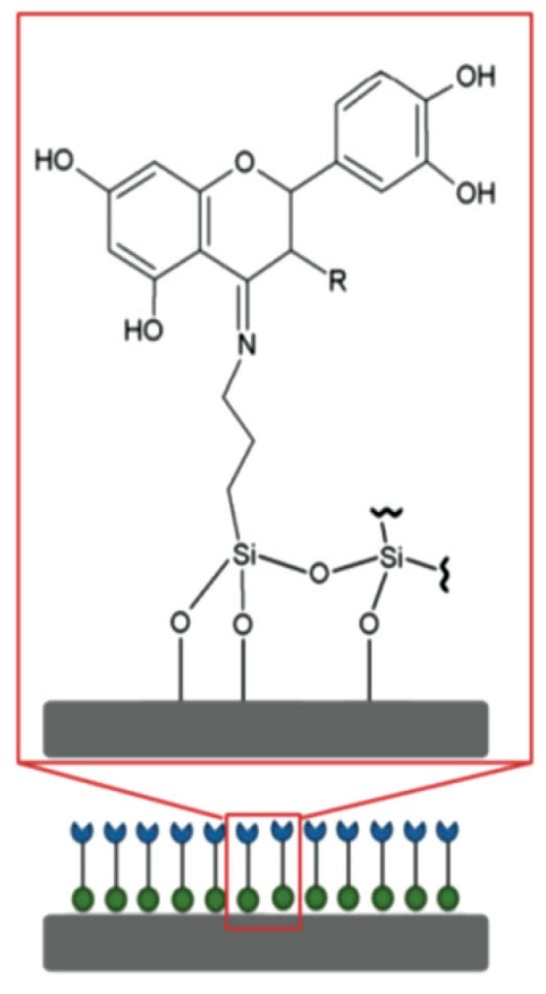
Fabrication of flavonoid-modified Ti surfaces. Flavonoids were covalently grafted to Ti through an APTES cross-linker. Reproduced from A. Córdoba et al. [168] with permission from John Wiley and Sons.

**Figure 11:**
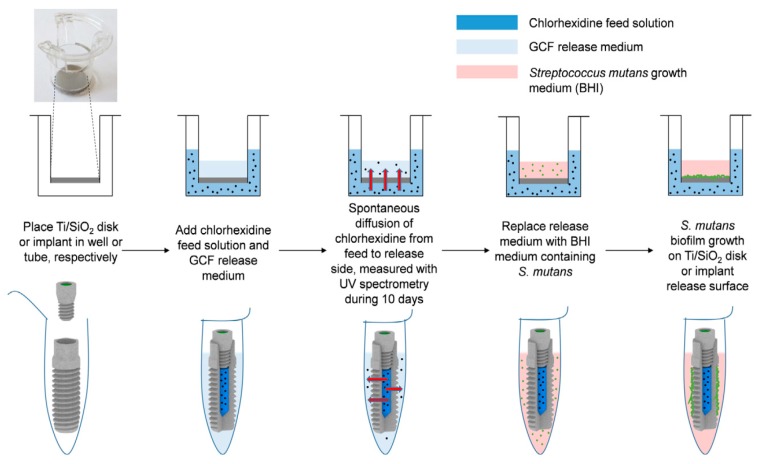
Schematic representation of the experimental setup of chlorhexidine release experiments and biofilm prevention using Ti/SiO_2_ disks or implants. The Ti/SiO_2_ material (grey disk or implant (upper and lower part, respectively)) is placed in its respective container. Chlorhexidine or control solutions (dark blue) and artificial gingival crevicular fluid (GCF medium, light blue) are administered to feed and release the compartments of both settings, respectively. The spontaneous diffusion of chlorhexidine from the feed to the release side is measured every 2 days using UV spectrophotometry. After 10 days, the GCF release medium is replaced with brain-heart infusion (BHI) medium (pink) containing approximately 2 × 10^6^ *Streptococcus mutans* cells/mL (green dots) to allow bacterial biofilm formation on the implant material (biofilm prevention). After 72 h, biofilm formation is quantified using metabolic staining and colony forming units (CFU) counting or visualized by SEM imaging. Reproduced from De Cremer, K., et al. [172].

Antibiotics can also be incorporated in the implant surface to selectively kill bacteria without side effects for eukaryotic cells. The main issue of implants loaded with antibiotics is the generation of antibiotic-resistant strains and the loss of bactericidal activity over time, which compromises the use of these agents. Tetracycline-loaded fibers obtained by electrospinning showed an excellent potential for killing peri-implantitis-associated pathogens and exhibited a total eradication of biofilm formation [165]. Additionally, other authors tested the osteogenic ability of this system and found that, besides the bactericidal capacity, the expression of the ALP kinase measured was remarkably higher than the control, promoting early bone formation [166]. Flavonoids are known for their good properties in biological processes, like antioxidant, anti-inflammatory, and anti-microbial capacity, among others. For this reason, the use of this active component in dental implantology has been studied in recent years. In particular, the functionalization of titanium surfaces with taxifolin and quercetin flavonoids has been addressed because they have been reported as osteogenic inducers and inhibitors of peri-implantitis [167]. Flavonoid immobilization was carried out with (3-aminopropyl) triethoxysilane (TESPA) as a coupling agent, and the results showed activation of osteogenesis mechanisms, anti-inflammatory, and anti-fibrotic properties, as well as effective inhibition of the development of peri-implantitis disease (Figure 10) [168]. Other authors loaded TNTs with propolis, which is a resinous natural product rich in flavonoids, and tested the in vitro and in vivo action, yielding high rates of cell proliferation and differentiation and correct osseointegration in a rat model [169]. Gentamycin has been employed as an antibacterial agent. It has been loaded into fibroin nanoparticles with and RGD peptides, obtaining a biologically active nanorough surface. These implants inhibited the growth of *Staphylococcus aureus* while promoting osteoblast adhesion and differentiation, as well as high deposition of CaP and an overexpression of ALP kinase [170]. Other authors coordinated gentamycin with Ag nanoparticles, which were reduced in situ by silk fibroins to form a stable complex with strong antibacterial activity and bone formation capacity [171]. Moreover, chlorhexidine has been used as an antibacterial agent loaded into microporous silica coatings by diffusion, avoiding the burst release of the drug and obtaining satisfactory antibacterial and osteogenic properties (Figure 11) [172]. Dopamine coatings can also be employed to covalently immobilize an antibiotic. The fixation of bacitracin has been reported in polydopamine-modified surfaces [173]. Antimicrobial tests against *Staphylococcus aureus* and methicillin-resistant *Staphylococcus aureus* (MRSA) showed a significant inhibition of bacteria colonization while the adhesion, proliferation, and osteogenic differentiation of o human bone marrow mesenchymal stem cells (hBMSCs) were higher than the control group without bacitracin. In addition, it was found that the secretion of pro-inflammatory cytokines was lowered due to the inhibitory action of bacitracin on macrophage spreading. This led to a better osseointegration process without side effects caused by surrounding inflamed tissues. Other authors have combined the use of polymers, like chitosan [174] and hyaluronic acid [175], as coatings with vancomycin-encapsulated TNTs. Chitosan and hyaluronic acid were functionalized with catechol, which can reduce the concentration of reactive oxygen species and therefore inflammation of the tissues, favoring the osteogenic activity of implants. Besides, the degradation of hyaluronic acid carried out by bacterial hyaluronidase [176,177] leads to a slow release of the bactericidal peptide. The excellent antibacterial potential showed by these functionalized devices permitted not only inhibition of *S. aureus* attachment in vitro but also increased the formation of new bone tissue when they were implanted in an in vivo infected defect [178]. Other authors have reported the functionalization of implants with alendronate as a promoting agent of bone growth by the self-assembly monolayer technique. This novel methodology allowed the formation of a uniform drug coating, which showed an excellent osteogenic differentiation of human mesenchymal stem cells (hMSC) [179].

### 3.4. Anti-Adhesive Coatings

The use of anti-adhesive polymers that inhibit the attachment of bacteria is another approximation described in the literature to obtain antibacterial and osteogenic implants [180]. The only disadvantage presented by these coatings is the unspecific suppression of osteoblasts and bacteria fixation, and therefore they must be functionalized with a peptide or a bioactive compound that enhances the adhesion of eukaryotic cells. Chitosan and carboxymethyl chitosan are the most extensive antimicrobial polymers employed. In this regard, publications have been found in which different osteogenic agents, such as hydroxyapatite [181], bone morphogenetic protein-2 (BMP-2) protein [182], ALP [183], silica–chitosan hybrid materials [184], and chitosan-58S bioactive glass nanocomposite [185], are included in the formulation to improve osteoblast response. All reports coincided in a complete inhibition of bacterial adhesion combined with an improved osteointegration obtained with this type of system. 

## 4. Conclusions

A consensus exists on the successful clinical outcomes for most commercial dental implants in the short and medium term, with some differences on the osteointegration degree depending on the manufacturing technique used. However, no high rates of significant improvements in the long-term performance were exhibited between modified surfaces and machine-turned implants, in which one of the most common causes of implant failure is the generation of periodontal disease as a consequence of implant colonization by orally present bacteria. In accordance with the literature reviewed in this article, the most promising research to ensure both osteogenic and anti-peri-implantitis functionality of the next generation of dental implants is focused on the development of bioactive surfaces by industrially reliable manufacturing techniques, such as acid etching, anodization, or laser ablation, which combine antimicrobial activity with osteogenic capacity to optimize healing and antibacterial capacities in the initial states and therefore achieve correct osseointegration and long-term stability. Nevertheless, an overview of the present review indicates that much work remains to be done on the development of new devices that ensures their long-term durability and capacity to prevent peri-implantitis disease.

## Figures and Tables

**Table 1:** Manufacturing techniques applying physico-chemical treatments used in commercial implants.

Manufacturing Technique	Example of Commercial Brand
Sandblasting	TiOblast^®^ (Astra Tech, Mölndal, Sweden), Swede and Screw Vent^®^ (Zimmer Biomet, Palm Beach Gardens, Florida, USA) and Standard, Hex^®^ (Osteoplant, Poznan, Poland).
Acid-etching	Osseotite^®^ (Zimmer Biomet, Warsaw, Indiana, USA) and Steri-Oss Etched^®^ (Nobel Biocare, Zürich-Flughafen, Switzerland)
Grit blasting and acid-etching	SLA Straumann^®^ (Straumann Institute, Basel, Switzerland), Ankylos^®^ (Dentsply Friadent, Mannheim, Germany), Friadent Plus^®^ (Dentsply Friadent, Mannheim, Germany), Promote^®^ (Camlog, Basel, Switzerland) and Osseonova^®^ (Ziacom, Pinto, Spain)
Anodization	TiUnite^®^ (Nobel Biocare, Gothenburg, Sweden)
Plasma spraying	IMZ-TPS^®^ (Dentsply Friadent, Mannhein, Germany), Bonefit^®^ (Straumann Institute, Waldenburg, Switzerland), Restore-TPS^®^ (Lifecore Biomedical, Chaska, Minnesota, USA), Steri-Oss-TPS^®^ (Nobel Biocare, Yorba Linda, California, USA) and ITI-TPS^®^ (Straumann Institute, Waldenburg, Germany)

**Table 2:** Nanorough surface-treated implants available in the market.

Manufacturing Technique	Examples of Commercial Brand
Laser Ablation	Laser-Lok^®^ (BioHorizons, Birmingham, Alabama)
Grit blasting and acid etching	SLActive^®^ (Straumann Institute, Basel, Switzerland), Osseospeed^®^ (Dentsply Friadent, Mannheim, Germany)

**Table 3:** Commercially available implants with inorganic surface coatings.

Coating Component	Examples of Commercial Brand
Hap	Osstem TS III-HA^®^ (Osstem Implant Co., Busan, Korea) and Osstem GS-HA III^®^ (Osstem Implant Co., Busan, Korea), TSV-HA^®^ (Zimmer Biomet, Carlsbad, California, USA)
CaP nanoparticles	Ossean^®^ (Intra-Lock, Boca Raton, Florida, USA)
CaP and DCD nanotopography	Nanotite^®^ (Zimmer Biomet, Palm Beach Gardens, Florida, USA)
Nanostructured Ca	XPEED^®^ (MegaGen Implant Co., Gyeongbuk, South Korea)

**Table 4:** Classification of surfaces presented by commercial dental implants according to their roughness * obtained by means of different manufacturing technique.

		Surface Modification Process				
	Commercial Brand	Sandblasting	Acid Etching	Anodization	Plasma Spraying	CDC
Smooth Surface (S_A_ = 0–0.5 µm)	Brånemark^®^					
Minimally Rough (S_A_ = 0.5–1 µm)	Osseotite^®^		×			
	Nanotite^®^	×	×			
Moderately Rough (S_A_ = 1–2 µm)	TiOblast^®^	×				
	Steri-Oss Etched^®^		×			
	SLA Straumann^®^	×	×			
	SLActive^®^	×	×			
	Osseospeed^®^	×	×			
Rough Surfaces (S_A_ > 2 µm)	Ankylos^®^	×	×			
	Friadent Plus^®^	×	×			
	TiUnite^®^			×		
	ITI-TPS^®^				×	
	TSV-HA^®^					×

* As classified by Wennerberg A, Albrektsson T. 2010 [130].
